# Deep Learning Analysis of Cardiac MRI in Legacy Datasets: Multi-Ethnic Study of Atherosclerosis

**DOI:** 10.3389/fcvm.2021.807728

**Published:** 2022-01-21

**Authors:** Avan Suinesiaputra, Charlène A. Mauger, Bharath Ambale-Venkatesh, David A. Bluemke, Josefine Dam Gade, Kathleen Gilbert, Markus H. A. Janse, Line Sofie Hald, Conrad Werkhoven, Colin O. Wu, Joao A. C. Lima, Alistair A. Young

**Affiliations:** ^1^Department of Anatomy and Medical Imaging, University of Auckland, Auckland, New Zealand; ^2^Department of Biomedical Engineering, School of Biomedical Engineering and Imaging Sciences, King's College London, London, United Kingdom; ^3^Johns Hopkins Medical Center, Baltimore, MD, United States; ^4^Department of Radiology, University of Wisconsin School of Medicine and Public Health, Madison, WI, United States; ^5^Department of Biomedical Engineering and Informatics, School of Medicine and Health, Aalborg University, Aalborg, Denmark; ^6^Auckland Bioengineering Institute, University of Auckland, Auckland, New Zealand; ^7^Department of Electrical Engineering, Eindhoven University of Technology, Eindhoven, Netherlands; ^8^Division of Intramural Research, National Heart, Lung and Blood Institute, National Institutes of Health, Baltimore, MD, United States; ^9^Faculty of Life Sciences & Medicine, School of Biomedical Engineering & Imaging Sciences, King's College London, London, United Kingdom

**Keywords:** cardiac anatomy, machine learning, left ventricle, MRI, deep learning

## Abstract

The Multi-Ethnic Study of Atherosclerosis (MESA), begun in 2000, was the first large cohort study to incorporate cardiovascular magnetic resonance (CMR) to study the mechanisms of cardiovascular disease in over 5,000 initially asymptomatic participants, and there is now a wealth of follow-up data over 20 years. However, the imaging technology used to generate the CMR images is no longer in routine use, and methods trained on modern data fail when applied to such legacy datasets. This study aimed to develop a fully automated CMR analysis pipeline that leverages the ability of machine learning algorithms to enable extraction of additional information from such a large-scale legacy dataset, expanding on the original manual analyses. We combined the original study analyses with new annotations to develop a set of automated methods for customizing 3D left ventricular (LV) shape models to each CMR exam and build a statistical shape atlas. We trained VGGNet convolutional neural networks using a transfer learning sequence between two-chamber, four-chamber, and short-axis MRI views to detect landmarks. A U-Net architecture was used to detect the endocardial and epicardial boundaries in short-axis images. The landmark detection network accurately predicted mitral valve and right ventricular insertion points with average error distance <2.5 mm. The agreement of the network with two observers was excellent (intraclass correlation coefficient >0.9). The segmentation network produced average Dice score of 0.9 for both myocardium and LV cavity. Differences between the manual and automated analyses were small, i.e., <1.0 ± 2.6 mL/m^2^ for indexed LV volume, 3.0 ± 6.4 g/m^2^ for indexed LV mass, and 0.6 ± 3.3% for ejection fraction. In an independent atlas validation dataset, the LV atlas built from the fully automated pipeline showed similar statistical relationships to an atlas built from the manual analysis. Hence, the proposed pipeline is not only a promising framework to automatically assess additional measures of ventricular function, but also to study relationships between cardiac morphologies and future cardiac events, in a large-scale population study.

## Introduction

Cardiovascular magnetic resonance (CMR) is widely used for the non-invasive assessment of cardiac function, and has excellent accuracy and reproducibility for clinical evaluation of cardiac mass and volume (1). The ability of CMR to evaluate all regions of the heart with high signal to noise ratio without harmful radiation exposure has led to its use in several large cohort studies investigating the development of cardiac disease in general populations, including the Multi-Ethnic Study of Atherosclerosis (MESA) (2) and the UK Biobank (3). MESA was the first large epidemiological study to utilize CMR to evaluate pre-clinical characteristics of participants before the onset of clinical symptoms of cardiovascular disease (CVD). The baseline MESA CMR exam was performed between 2000 and 2002 using the common imaging method prevalent at that time: gradient echo cine imaging. However, this imaging method has been largely replaced by steady-state free precession cine imaging in subsequent studies and in clinical practice (4). Due to differences in fundamental properties that comprise image contrast as well as spatial resolution (5), image analysis tools designed for modern steady-state free precession images are likely to have poor performance when applied to 20-year-old gradient echo imaging.

Three-dimensional (3D) atlas-based analysis methods have been developed to quantify subtle differences in heart shape (remodeling) and function associated with CVD risk factors such as hypertension, smoking and diabetes (6–10). To date, these methods have only been applied to a limited subset of MESA cases, due to the need for additional image analysis which was not performed as part of the original CMR analysis. This is a recurring problem in large cohort legacy datasets, since a limited amount of annotations are available and manual analysis is unfeasible due to time and resource constraints. A fully automated processing pipeline is therefore necessary to enable more comprehensive analysis and make better use of the large amount of image data acquired.

Deep learning methods, particularly convolutional neural networks (CNN), have demonstrated high accuracy and reproducibility for fully automated image analysis when sufficient training images and high computational power is available (11, 12). CNN can automatically learn optimal weights for convolutional operations in each layer to extract image features. It has been applied and adapted for image classification (13), object recognition (14), segmentation (15), and image registration (16). However, CNN solutions trained on modern steady-state free precession images fail when applied to the old gradient echo images. Transfer learning approaches, such as pre-training or layer-wise fine tuning, have been proposed to adapt a network to different domain, but when large amount of labeled data is available, full training from scratch is the best option to train a CNN (17).

**Figure 1:**
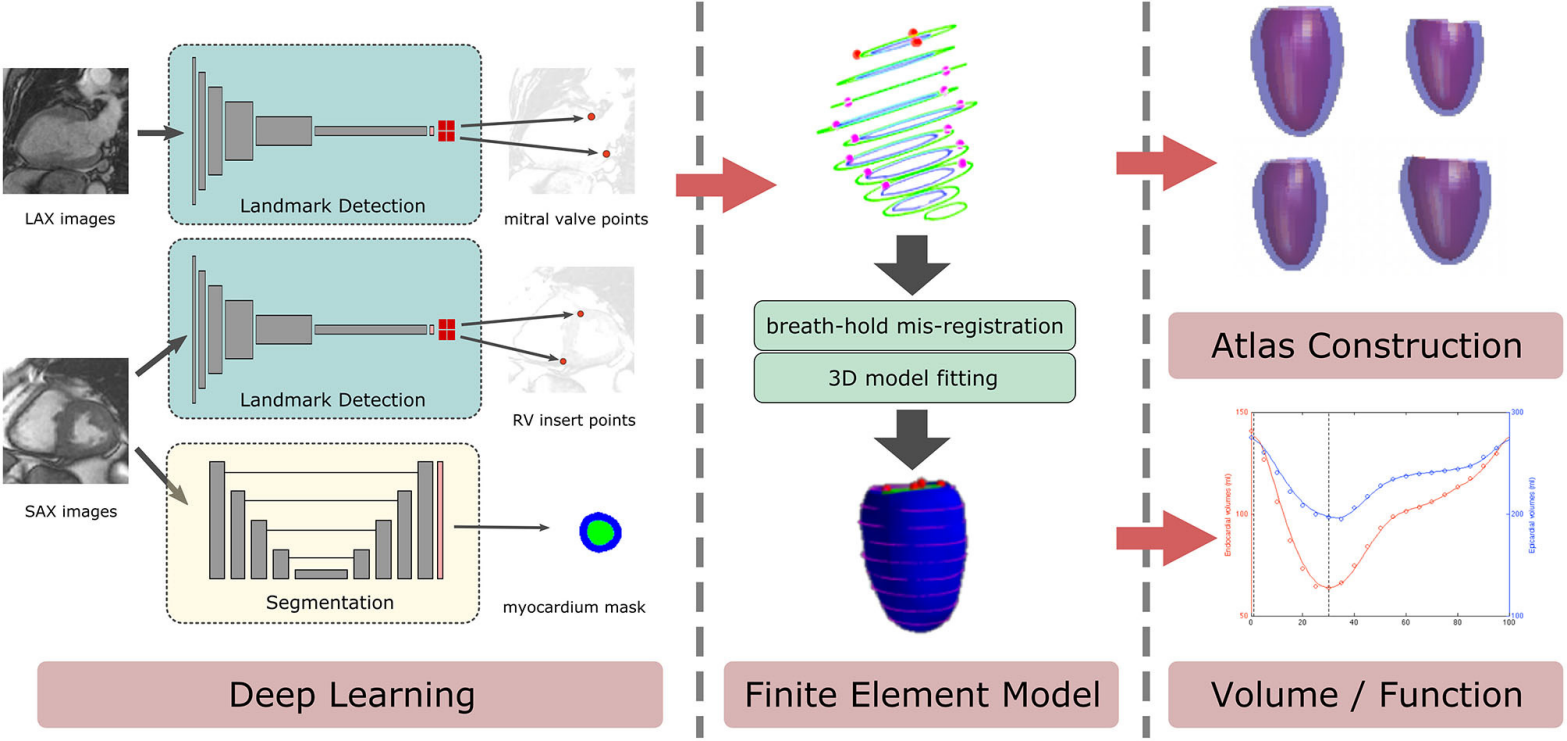
Fully-automated atlas generation pipeline of cardiac MRI analyses. Three deep learning networks were trained to perform: (1) detection of mitral valve points from long-axis (LAX) images, from both two-chamber or four-chamber views, (2) detection of right ventricular (RV) insert points from short-axis (SAX) images, and (3) segmentation of myocardium mask from SAX images. Landmark points and contours from myocardium mask images were converted into 3D patient coordinates to guide the customization of a left ventricle (LV) model. Breath-hold mis-registration of SAX slices were corrected. The final model was used to construct a statistical shape LV atlas.

In this study, we developed an automated CMR preprocessing pipeline, shown in Figure 1. In order to automatically construct 3D LV shape models and a statistical shape atlas, anatomical landmarks were required to orient the model and contours were required to customize the shape models. Custom CNNs were used to detect anatomical landmarks and to segment myocardium from the MESA gradient echo CMR images. We demonstrate that these networks provide robust and consistent contours and landmarks compared with manual annotations. We also show that an LV atlas built from the proposed pipeline produced similar associations with CVD risk factors to an atlas built from manual analyses.

## Materials and Methods

### Dataset

The MESA study has been described previously in (2). Briefly, the CMR exam consisted of 5,098 participants who were initially free from clinically recognized CVD at the time of enrollment (18). Images were acquired with 1.5T MR scanners at six different institutions across the United States using Siemens and General Electric scanners between July 2000 and July 2002. All images were acquired during breath-holding at resting lung volume. From each CMR examination, we only included short- and long-axis cine images for this study. The cine CMR images consist of 10–12 short-axis slices (SAX), single four-chamber (4CH) and single two-chamber (2CH) long-axis (LAX) views. All cine images were acquired using fast gradient echo pulse sequence, with typical parameters of slice thickness 6, 4 mm gap, field of view 360–400 mm, 256 × 160 image matrix (smallest 192 × 160), flip angle 20°, echo time 3–5 ms, repetition time 8–10 ms with 20–30 frames per slice (temporal resolution <50 ms) and pixel size from 1.4 to 2.5 mm/pixel depending on patient size. All participants gave informed consent, and the institutional review board at each site approved the study protocol.

The MESA Core Lab provided 2D contour points drawn manually by trained technologists. The Core Lab analysis protocol for MESA study has been described previously (18), including inter- and intra-observer variability. Briefly, endocardial and epicardial borders were traced on short-axis slices at end-diastole (ED) and end-systole (ES) frames using Q-MASS software (version 4.2, Medis, the Netherlands). Papillary muscles were included in the blood pool. All image contours were reviewed and corrected by a cardiac MR physician.

**Figure 2:**
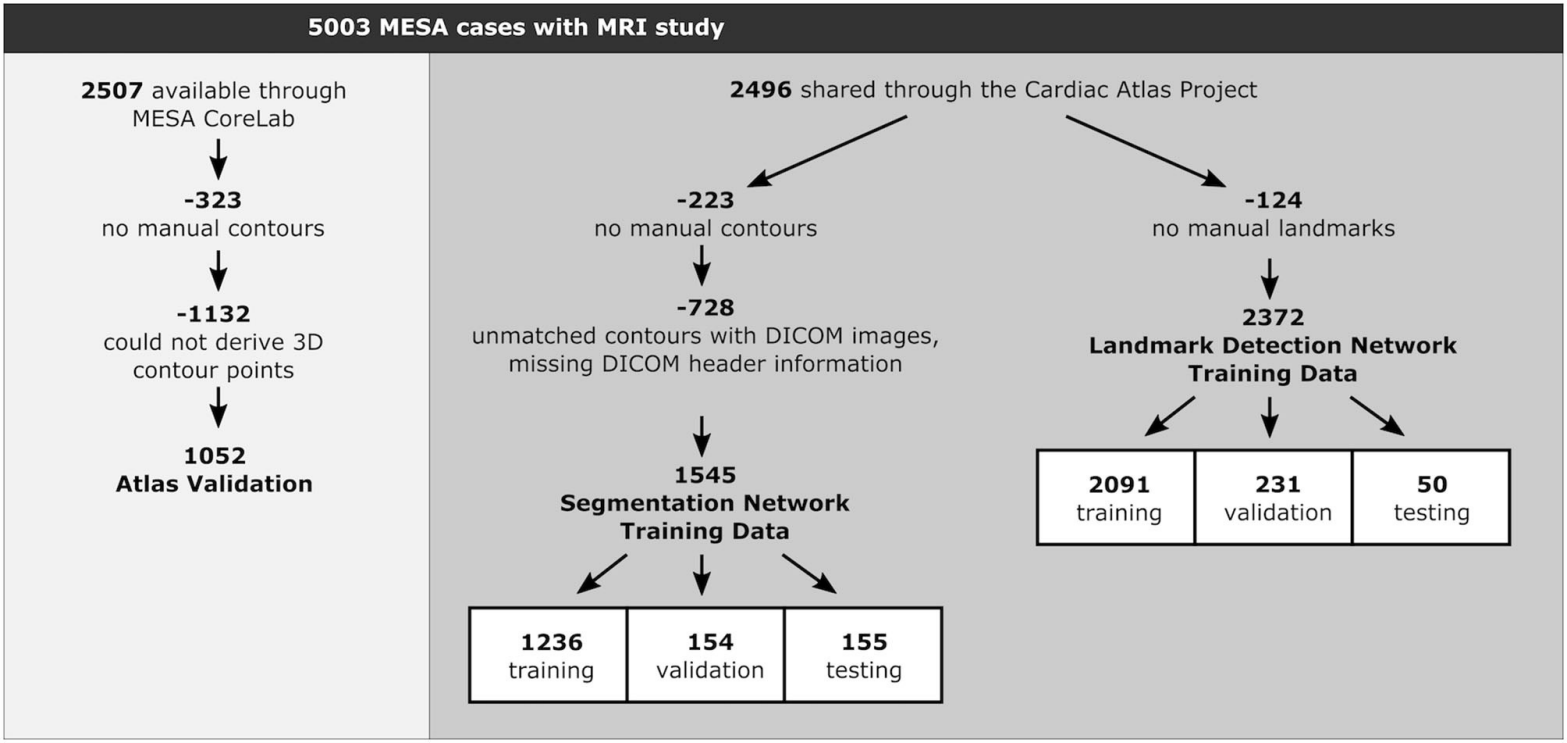
Division of MESA cases into two independent sets of Atlas Validation and Training sub-cohorts. Within the Training sub-cohort, cases were divided into training, validation and testing sub-groups for the different deep learning networks (Segmentation Network and Landmark Detection Network).

In total 5,003 exams had adequate MRI data for analysis (Table 1). Of these, 2,496 cases (49.9%) were available from the Cardiac Atlas Project (19), while the remaining 2,507 cases (50.1%) were provided by the MESA Core Lab at the Johns Hopkins Medical Center, Baltimore, USA. In this study, we used cases from the Cardiac Atlas Project for training, testing and validating the deep learning networks, while the remaining cases were used for an independent LV atlas validation. Figure 2 shows detail divisions of the baseline MESA cohort for the automated CMR analysis pipeline development.

**Table 1:** Patient demographics from the MESA cohort.

		**MESA CMR**	**Landmark detection**	**Segmentation**	**Atlas validation**
*N*		5,003	2,372	1,545	1,052
Age (years)		61.5 (10.1)	61.3 (10.1)	61.0 (10.2)^**^	60.1 (9.8)^***^
Gender	Female	2,622 (52.4)	1,230 (51.9)	814 (52.7)	430 (40.9)^***^
	Male	2,381 (47.6)	1,142 (48.1)	731 (47.3)	622 (59.1)
SBP (mmHg)		125.4(21.3)	126.2 (21.9)^*^	126.4 (22.0)^*^	124.8 (20.2)
DBP (mmHg)		71.8 (10.30)	71.6 (10.3)	71.7 (10.3)	73.6 (10.1)^***^
Heart Rate (bpm)		62.8 (9.5)	62.7 (9.5)	62.9 (9.5)	62.1 (9.6)^**^
Diabetes	Yes	459 (9.2)	232 (9.8)	162 (10.5)^*^	74 (7.0)^**^
	No	4,544 (90.8)	2,140 (90.2)	1,383 (89.5)	978 (93.0)
Hypertension	Yes	1,766 (35.3)	805 (34.0)	539 (34.9)	373 (35.5)
	No	3,234 (64.7)	1,566 (66.0)	1,005 (65.1)	677 (64.5)
Smoking status	Never	2,569 (51.5)	1,237 (52.3)	805 (52.4)	511 (48.6)
	Former	1,786 (35.8)	824 (34.9)	521 (33.9)	394 (37.5)
	Current	634 (12.7)	302 (12.8)	209 (13.6)	146 (13.9)
Framingham score		13.9 (9.5)	14.1 (9.5)	14.0 (9.6)	13.7 (9.2)

*Two sub-cohorts were defined to train and validate deep learning networks for landmark detection and segmentation. Another sub-cohort, disjoint from the two training datasets, was defined for validation of the atlas generated from automated compared with core lab manual analysis. Continuous variables are written as mean (standard deviation), while categorical variables are written as count (percentage). Statistical tests were performed between a sub-cohort against its complement with one-way ANOVA for continuous variables and **χ**^**2**^ test for categorical variables*.

* *p < 0.05*,

** *p < 0.01*,

*** *p < 0.001 for difference between a particular sub-cohort and the rest of the MESA CMR cohort*.

Of the 2,496 cases for the training data, 2,273 cases had manual contours. We further excluded 728 cases due to mis-alignment of contours with the image slices, unmatched contours with DICOM images or missing DICOM header information. This resulted in 1,545 cases to train the segmentation network, which were randomly split into 1,236 training cases (80%), 154 validation cases (10%), and 155 test cases (10%). Contour points were converted into mask images consisting of three disjoint areas: myocardium, LV cavity, and background pixels.

As anatomical cardiac landmark points were not part of the MESA Core Lab protocol, we employed two experienced analysts (both had >5 years of fulltime experience in CMR exams) to manually place cardiac landmarks by using Cardiac Image Modeler software (version 6.2; Auckland MR Research Group, University of Auckland, New Zealand). Of the 2,496 cases for the training data, 2,372 cases had adequate annotations to train the landmark detection network. These were randomly split into 2,091 training cases (88%), 231 validation cases (10%), and 50 test cases (2%). The test cases were also used for inter-observer variability study, where landmark points from both analysts are available for each case.

For the LV atlas validation, we need cases where we can derive 3D points from the manual contours. Unfortunately, information about 3D image positions and orientations were not stored in the Q-MASS contour files available from the Core Lab. We therefore developed a simple matching algorithm to align Q-MASS contours with the DICOM image headers. This consisted of ordering the images and contours from apex to base, followed by alignment based on image position and orientation. The alignment results were manually reviewed to confirm correct matching of contours and images. This process resulted in 1,052 cases with manually verified DICOM image matching, sufficient to validate the automated pipeline developed in this study (see Figure 2).

### Cardiac MRI Analysis Pipeline

As shown in Figure 1, the proposed automated CMR analysis combines two types of CNNs (myocardial segmentation and landmark detection) with LV finite element shape modeling. Cardiac landmark points were needed to determine the initial pose and orientation of the LV model, but were not part of the original MESA analysis protocol, hence further annotation was required to provide training data. The LV contours were required to guide the patient-specific customization of the LV model, and training data could be provided from the original MESA CMR analyses.

#### Landmark Detection Network

The landmark detection network was based on the VGGNet architecture (20), which has been successfully used to classify images and to recognize objects. It consists of 16 layers of CNN blocks that gradually extract features into smaller tensor size. The input is 256 × 256 MR image and the output is a feature vector of 2,048 elements. The final layer reduces this feature vector into four neurons corresponding to two points on the input image in [*x*_1_, *y*_1_, *x*_2_, *y*_2_] format. Details of this landmark detection architecture are given in Appendix A.

Two types of anatomical landmarks are predicted for the proposed pipeline. The first landmark is the position of mitral valve hinge points at the intersection between the left atrium and the left ventricle from two long-axis MR images: two-chamber (2CH) and four-chamber (4CH) views. The other landmarks are the position of the intersection points between the right ventricle and the interventricular septum (RV insert points) from short-axis MR images. Mitral valve points were used to determine the basal extent of the heart, whereas RV insertion points were used to estimate the position of the septum.

Although sharing the same architecture, we trained three separate landmark detection networks to detect the different types of cardiac landmark points and image views: 2CH mitral valve points, 4CH mitral valve points and short-axis RV insert points. We developed a novel transfer learning scheme between these networks during training, which was designed to exploit similarities in the images, yet allowing for differences in the spatial relationships. First, an initial network for one view was trained from scratch with random weight initialization until convergence. Then, the network was retrained for one of the remaining two views. However, instead of using a random initialization, the weights from the previous training step were used as initial weights. After the new network was converged, its weights were used as initialization for the third view. The order in which the three different views were trained was random. This sequence was repeated until convergence (e.g., the performance of the two-chamber network compared to the previously trained two-chamber network was not improved). An advantage of this sequence is that it allows for maximum freedom when training the neural networks for the different kinds of image views whilst still being able to infer features learned from other images. Table 2 shows the improvement of performance using the transfer learning scheme in the validation set, where the landmark distance errors significantly decreased.

**Table 2:** Landmark distance errors from neural networks trained independently compared to networks trained with our training strategy.

	**System trained with independent neural networks**	**System trained with our training strategy**
Two-chamber	2.98 (1.44)	1.53 (0.74)
Four-chamber	3.24 (1.55)	1.44 (0.74)
Short axis	2.94 (1.6)	2.07 (1.11)

*Error values were measured on 232 validation cases and shown as mean (standard deviation). All values are in millimeters*.

On average, we included five frames per case for the mitral valve points on each of the four-chamber and two-chamber views, and five short-axis slices per case for the RV inserts on the end-diastolic frame. In total, there were 11,604 images for the two-chamber view, 11,670 images for the four-chamber view, and 13,402 images for the short-axis view. Images were whitened by subtracting the mean pixel intensity and divided by standard deviation, on a per-image basis. Zero-padded cropping was performed to create 256 × 256 input images as needed.

We validated the predicted landmark points by the Euclidean distance (in mm) on the image space. The strength of agreement between the landmark detection and the two analysts was measured using the intraclass correlation coefficient (ICC) with a two-way random effects model (21). A high ICC (close to 1) indicates a high similarity between landmark point locations from all observers.

#### Segmentation Network

To segment the myocardium, we used the U-Net architecture (22), which has been successfully used in a wide range area of medical image analysis (12). The input is 256 × 256 short-axis MR image and the output is a mask image of the same size that consists of either myocardium, cavity or background pixel. The short-axis image was segmented individually; no temporal or other spatial multi-slice information was learned for this segmentation network. During training, data augmentation was performed by image flipping, zoom, brightness, and contrast variations. Input images were zero-padded and cropped into 256 × 256 image size as needed. More details about the segmentation network architecture and its training results are given in Appendix B.

We validated the accuracy of the segmentation network by using the Dice score (23), for both myocardium and LV cavity. We also validated standard clinical measurements for post-processing CMR exams (1), which include LV volumes at end-diastole and end-systole, ejection fraction and LV mass. Volumes were estimated by the LV cavity areas times the slice thickness (and slice gaps) for all short-axis slices where endocardial contours were available. LV masses were calculated from the myocardial volume (defined between endocardial and epicardial contours) multiplied by a density of 1.05 g/mL. All volumes and masses were indexed by body surface area, resulted in LV end-diastolic volume index (LVEDVi), LV end-systolic volume index (LVESVi), LV mass index (LVMi). Ejection fraction (LVEF) was measured by (LVEDVi – LVESVi) / LVEDVi ^*^ 100. We compared all these values from the test cases (*n* = 155) using the Bland-Altman plot analysis (24) to identify if there is a systematic error from the mean offset of the differences, inconsistent variability from the limits of agreement (mean ± 1.96 × standard deviation), and any trend of proportional error.

#### LV Atlas Construction

After landmark detection and segmentation (Figure 1), a finite element LV model was automatically customized to each set of myocardial contours and landmark points, as described previously in (25). Briefly, the LV model was first fitted to the landmark and contour points by a least squares optimization. The extent of the LV was defined from landmarks on mitral valve points and an LV apex point obtained from the contours. The septum area was located using the RV insertion landmark points. After orienting the model according to the landmarks, the endocardial and epicardial surfaces were fitted to the short axis contours by minimizing the distance between the surfaces and the contour points.

One advantage of using this LV model customization is that we can automatically correct image slice shifting due to breathing motion. In Figure 1, an example of this shifting artifact can be seen from the 3D contour points. The automatic breath-hold misregistration correction was based on (6). Briefly, a highly regularized customization of the LV was performed first to align a smooth LV model with the data. This model preserves the overall shape but is robust to breath-hold misalignments. Intersections between the LV model with short-axis image slices were then calculated and the contours were aligned with the model. The alignment movement was performed in-plane allowing only two degrees of freedom during shifting (no shift in the longitudinal direction). The shifting direction was calculated from the centroid of the intersection of the model with the image slice, based on the area-weighted average of the mesh barycenter. Then the LV model was re-customized to the data with a low regularization weight, minimizing the distance between the model and the contours.

After model fitting, an LV atlas was constructed by concatenating LV models from end-diastolic (ED) and end-systolic (ES) frames to capture both shape and motion information. In our previous study (7), concatenating ED and ES surface sample points yielded better performance to extract cardiac shape remodeling features compared to points from individual frames alone. Let *N* be the number of points sampled from the finite element model, and $P_{endo\text{\_}ED},\;P_{epi\text{\_}ED},P_{endo\text{\_}ES},\;P_{epi\text{\_}ES}\in {\mathbb{R}}^{Nx3}$ be 3D surface sampling points from the endocardium at ED, epicardium at ED, endocardium at ES and epicardium at ES, respectively. A single shape vector is defined by flattening each point matrix into ${S=\left[x_{1},y_{1},z_{1},\ldots ,x_{N},y_{N},z_{N}\right]}^{T}$vector and concatenating all of the four surfaces, resulting in 4 × 3 × *N* = 12*N* points. We removed position and orientation variations between shape vectors by using Procrustes alignment (26). The mean shape was then calculated and the principal component analysis (PCA) can be applied to the registered shape vectors.

### Association With Cardiovascular Risk Factors

To demonstrate the clinical efficacy of the predicted LV atlas, we analyzed associations between LV shape and cardiovascular risk factors, i.e., hypertension, diabetes, smoking status, cholesterol level, and calcium score, and compared atlas associations obtained from the automatic pipeline with atlas associations obtained from manual contours and landmarks. For this evaluation, we evaluated 1,052 MESA cases independent of the sub-cohorts used to train the landmark and segmentation networks (the atlas validation dataset, Table 1 and Figure 2).

Our hypothesis was that there is no significant differences in the strength of risk factor associations between the automatically generated LV atlas and the atlas derived from manual analyses. Logistic regression (LR) models were used to evaluate the strength of the risk factor associations. A separate LR model was generated for each risk factor using that factor as a binary univariate dependent variable and the first 20 principal component scores (90% total variance explained) derived from the atlas as the independent variables. Visual comparisons between modes of shape variations from LV Atlas derived from manual analyses and from the proposed cardiac MRI pipeline are available in the Supplementary Files. The strength of the association between shape and risk factor was quantified using the area under the curve of the receiver operating characteristic (AUC). To avoid overfitting, a ten-fold cross validation scheme was employed. At each cross validation iteration, we rebuilt the PCA from scratch to show that the associations were not dependent to a fixed orientation of the principal axes.

## Results

### Landmark Detection

The total training time for three landmark detection networks was 14 h on NVidia Titan X Pascal GPU. Typically, five iterations of transfer learning between 2CH, 4CH, and SAX networks were required for overall convergence. The performance of the landmark detection networks was tested on 50 independent cases, which were annotated by two expert analysts independently. Only images where both analysts identified all landmark points were included. These resulted in 111 2CH, 107 4CH, and 286 SAX images for comparisons. Since two points are identified from each image, the total number of points during the test was 222, 214 and 572 points for 2CH, 4CH, and SAX, respectively.

**Figure 3:**
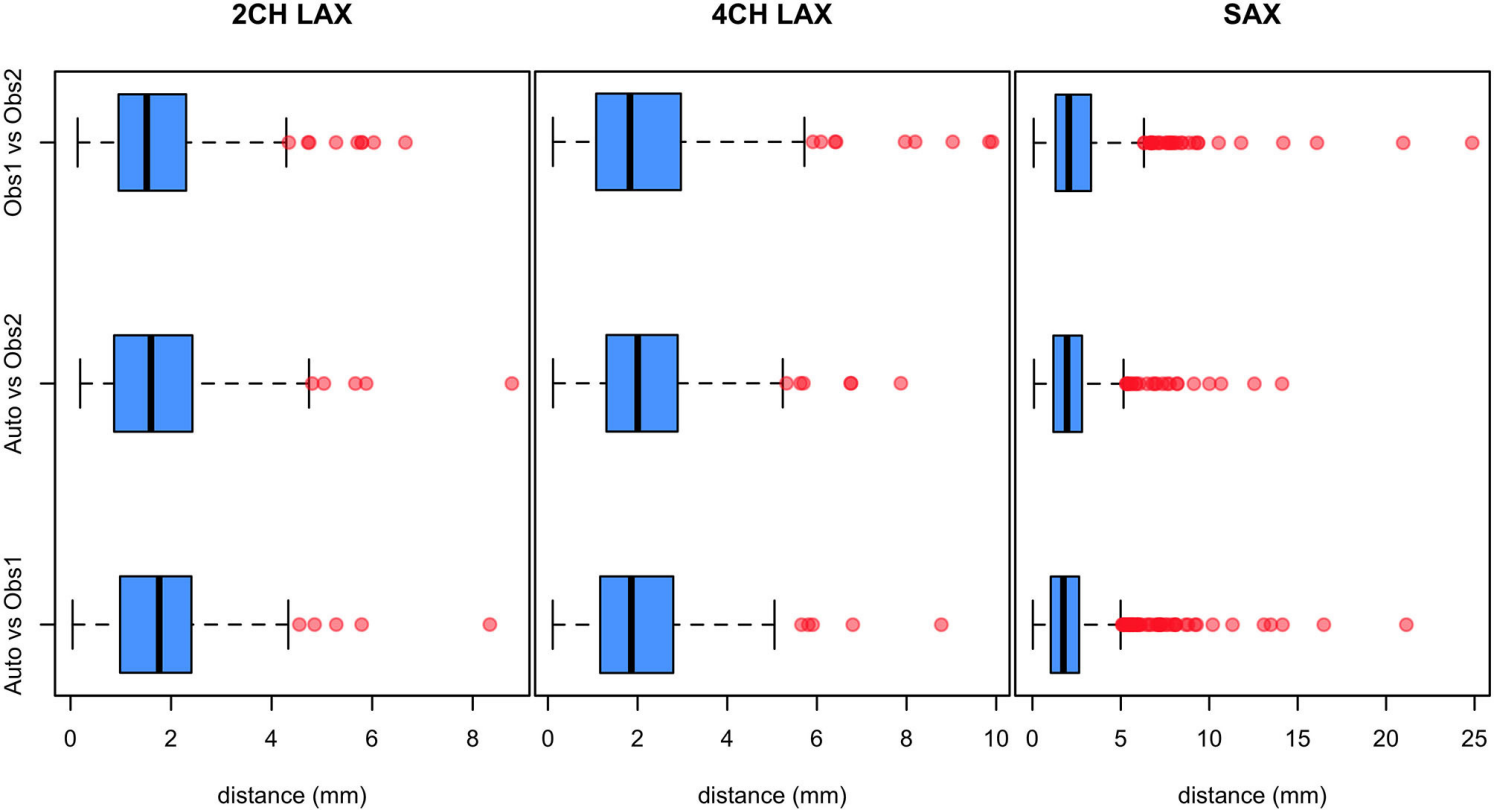
Distributions of distances between landmark points identified by the landmark detection method (Auto) and the two analysts (Obs1 and Obs2). Median (solid line), quartiles (thin lines) outliers (red points).

The distributions of Euclidean distances between automated methods and the observers are shown in Figure 3. Mean, standard deviation, and maximum distances are given in Table 3. The results show that the automated landmark detection errors are within the inter-observer variabilities with no significant differences in the location of landmark points (all *p* < 0.001). ICC between the automated method and the two analysts were all excellent, i.e., 0.998, 0.996, and 0.995 for 2CH, 4CH, and SAX respectively.

**Table 3:** Differences and intraclass correlation (ICC) values in detecting landmarks on 50 validation cases.

	**2CH LAX**	**4CH LAX**	**SAX**
	***N =* 222**	***N =* 214**	***N =* 572**
Auto vs. Obs1	1.86 (1.19)	2.09 (1.32)	2.29 (2.15)
Auto vs. Obs2	1.81 (1.21)	2.19 (1.28)	2.27 (1.61)
Obs1 vs. Obs2	1.78 (1.16)	2.24 (1.68)	2.67 (2.29)
ICC value	0.998	0.996	0.995

*All difference values are expressed mean (standard deviation) from the Euclidean distance between annotations in millimeters. N is the number of cases*.

**Figure 4:**
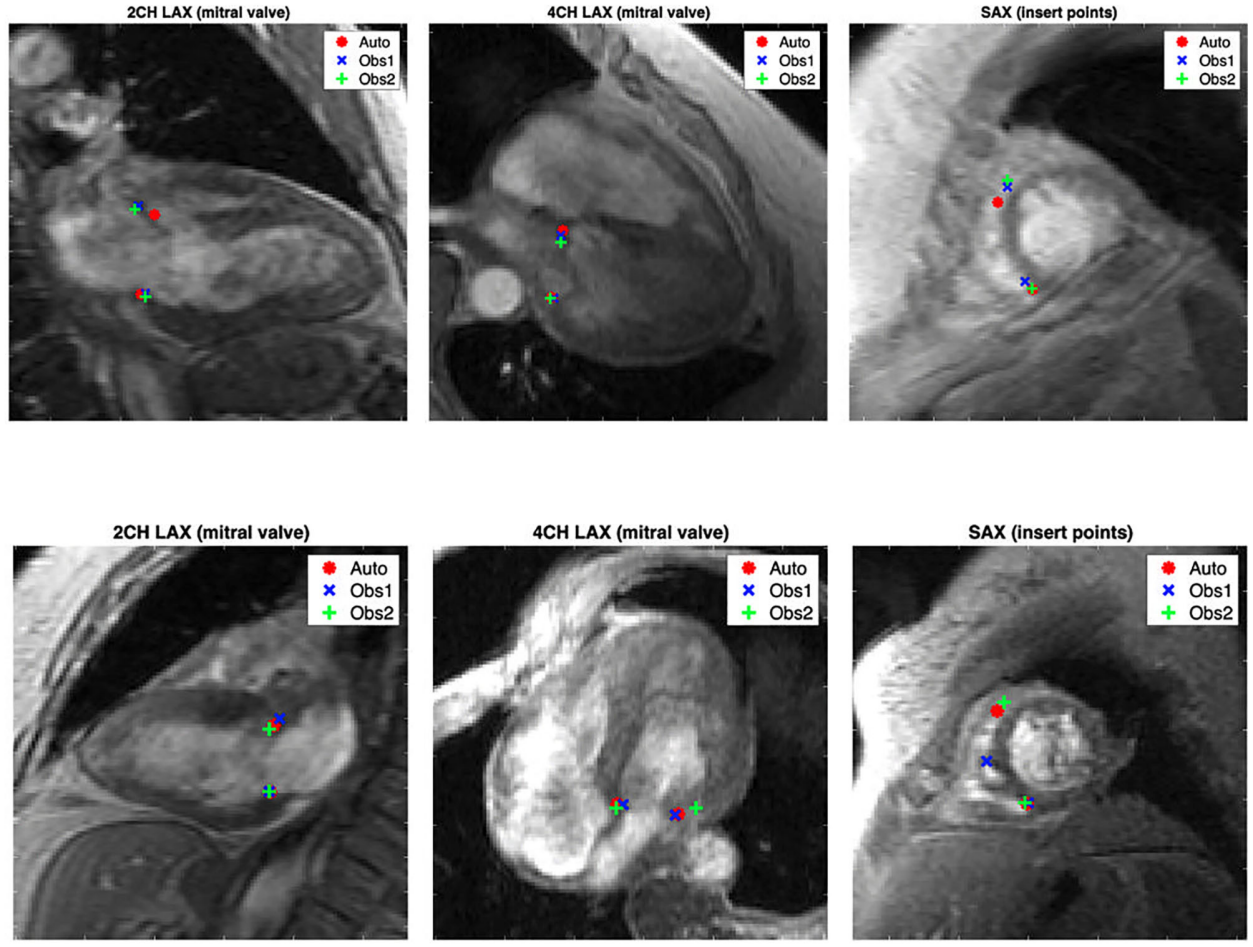
Examples of automated landmark detection (red markers) compared with manually defined placements by two observers (blue and green markers). The top row shows cases with the maximum distance of automated detection to one of the observers while interobserver distances are small. The bottom row shows cases with the largest interobserver distances.

Examples of landmark detections are shown in Figure 4 together with manual expert observer placements. The top row images show the largest distance of the automated detection method where the distance between observers was low (<3 pixels). Even in these cases, the automated method could identify the landmarks very close to the observers. The bottom row images in Figure 4 showcase the largest distances between expert observers. The automated method was able to identify landmark points in these cases with the position very close to one of the observers. These cases show the difficulty of visually identifying landmark points where image contrast is low and high image noise is present.

### Segmentation

**Figure 5:**
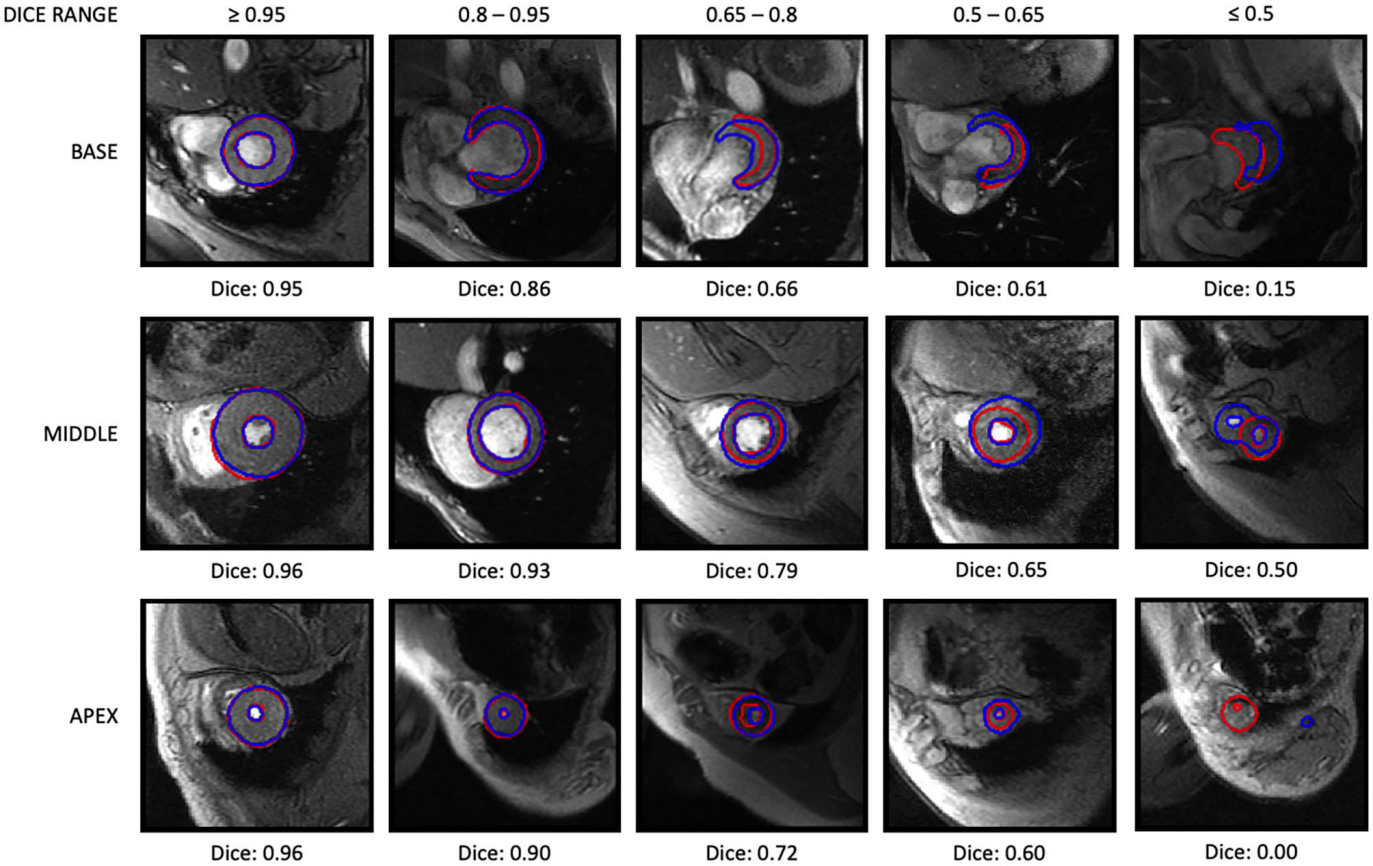
Examples of short axis segmentation network results. Top row, base; middle row, mid-ventricle; bottom row, apex. Manual contours are in red while automated contours are in blue. A range of Dice score results are shown.

Quartiles, means, and standard deviations of the Dice score from the test dataset are presented in Table 4. Median and mean Dice scores were high (>0.8) for myocardium and LV cavity masks, both at ED and ES frames. Typical segmentation results are shown in Figure 5 with cases of best, mean, and worst results. Figure 5 also demonstrates the difficulty of segmenting basal slices near the LV outflow tract.

**Table 4:** Dice score results of the segmentation network from the test dataset with 2,465 images.

**Mask**	**Frame**	**Q1**	**Median**	**Q3**	**Mean**	**Std dev**
Cavity	ED	0.92	0.95	0.97	0.93	0.07
	ES	0.86	0.91	0.94	0.88	0.11
Myocardium	ED	0.85	0.89	0.91	0.87	0.07
	ES	0.89	0.92	0.94	0.90	0.08

*Frames indicate end-diastole (ED) and end-systole (ES). The 25th quartile (Q1), median, and 75th quartile (Q3) are shown, together with means and standard deviations*.

**Figure 6:**
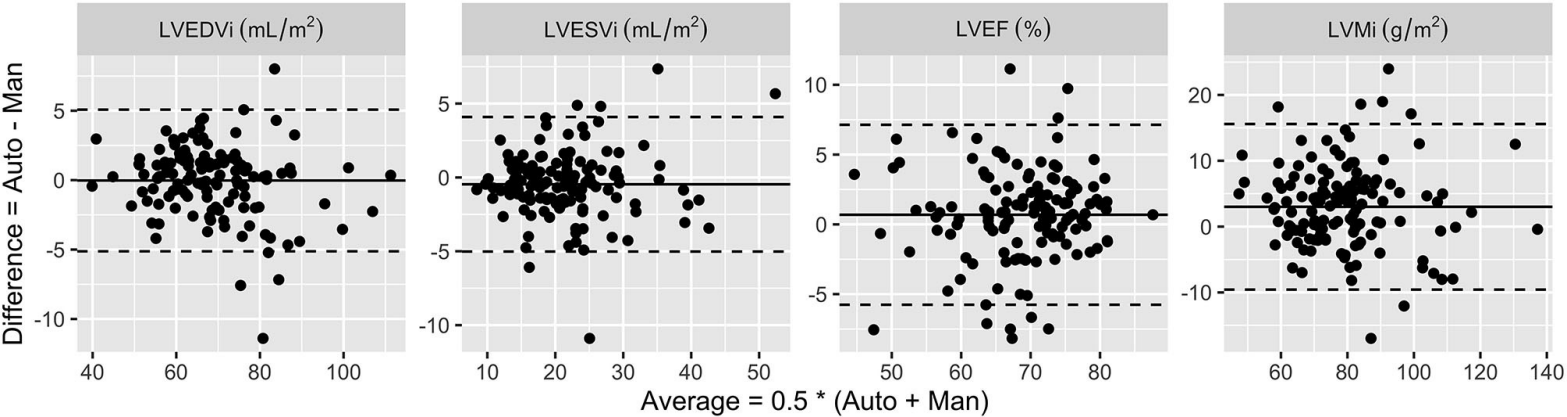
Differences between automated analysis (Auto) and manually drawn contours (Man). Solid lines are mean differences and dashed lines are the limits of agreement within ±1.96 × standard deviation from the mean. The mean difference values are shown in Table 5.

Table 5 shows comparisons of volumes (LVEDVi and LVESVi), mass (LVMi) and ejection fraction (LVEF) from the test cases. The segmentation network achieved excellent correlation coefficients for all clinical measurements (all Pearson's coefficients are >0.9, *p* < 0.001). The mean offset of differences are also small, i.e., <1 mL/m^2^ for volumes, only 0.7% for ejection fraction, and 3 g/m^2^ for mass. As shown in Figure 6, the differences are consistent within the limit of agreement lines without any visible trend for proportional error.

**Table 5:** Comparisons of indexed LV volumes, ejection fraction and mass from the 155 test cases between the predicted segmentation results with manual contours.

**LV function**	**Correlation coefficient**	**Differences**
LVEDVi (mL/m^2^)	0.98 (*p < * 0.001)	−0.02 (2.6)
LVESVi (mL/m^2^)	0.95 (*p < * 0.001)	−0.46 (2.3)
LVEF (%)	0.92 (*p < * 0.001)	0.69 (3.3)
LVMi (g/m^2^)	0.92 (*p < * 0.001)	3.0 (6.4)

*The differences are written as mean (standard deviation)*.

### Atlas Validation

Finally, we compared cardiovascular risk factor associations from the LV atlas from the automated analysis pipeline with an atlas formed from the manual analyses using a similar analysis method to (25). Table 6 shows the comparison of the area under the receiver operating characteristic curves (AUC) from risk factor association results (test cases from the cross validation). From all risk factors (hypertension, diabetes, smoking status, cholesterol, and calcium score), none of them have significant differences between the two methods except for cholesterol (*p* = 0.02) which showed a stronger association with the automated analysis than with the manual analysis.

**Table 6:** Area under the ROC curve (AUC) comparisons from the 1,052 LV shape association studies using different contours: manual (Man) and deep learning (Auto).

	**AUC**		***P*-value**
	**Man**	**Auto**	
Hypertension	0.69	0.71	0.22
Diabetes	0.56	0.53	0.34
Smoking status	0.59	0.61	0.33
Cholesterol	0.50	0.54	0.02
Calcium score	0.61	0.61	0.99

## Discussion

In this study, we present methods for the automated analysis of large cohort data from a legacy dataset obtained in the MESA study, aided by deep learning methods. These methods enable a more complete analysis of large cohort datasets, augmenting the parameter set available from these valuable studies. In addition to the end-diastolic and end-systolic volumes computed in the original study, these methods enable the analysis of 3D shapes, facilitating a fully automated 3D model-based atlas analysis method. Almost all risk factors showed similar strength of relationships with atlas scores, except for cholesterol level in which the automated method showed a stronger relationship (Table 5). However with AUC around 0.50, the elevated cholesterol association was essentially random. The slightly higher AUC for the automated contours may indicate that some signal may be available in the automated analysis which was lost in the manual analysis. This requires more research using a larger cohort.

The automated landmark detection method was successfully applied to GRE images, which are known to have lower signal-to-noise ratio and lower contrast compared to the current standard steady state free precession CMR imaging methods (5). The agreements with two expert analysts were all excellent (ICC > 0.9). Since signal-to-noise ratio is low in some gradient echo images, the analysts had noticeable disagreements between them in some cases, as shown in Figure 4 (bottom row). However, the automated detection method could identify the location of the landmark point in agreement with one of the observers. This ability was achieved by our approach to transfer learning weight parameters between image views iteratively. We exploited features between different domains to make the detection robust to noise and other artifacts.

Other machine learning methods have reported good results with landmark detection in cardiac MRI data, as well. For instance, Tarroni et al. (27) applied a hybrid random forest approach integrating both regression and structured classification networks and reported mean errors of 3.2–3.9 mm in mitral valve landmark detection. Although it is difficult to determine which methods give the “best performance” in this application, our results show that the CNN-based method is powerful enough in the applications where legacy datasets provide sufficient annotated cases.

**Figure 7:**
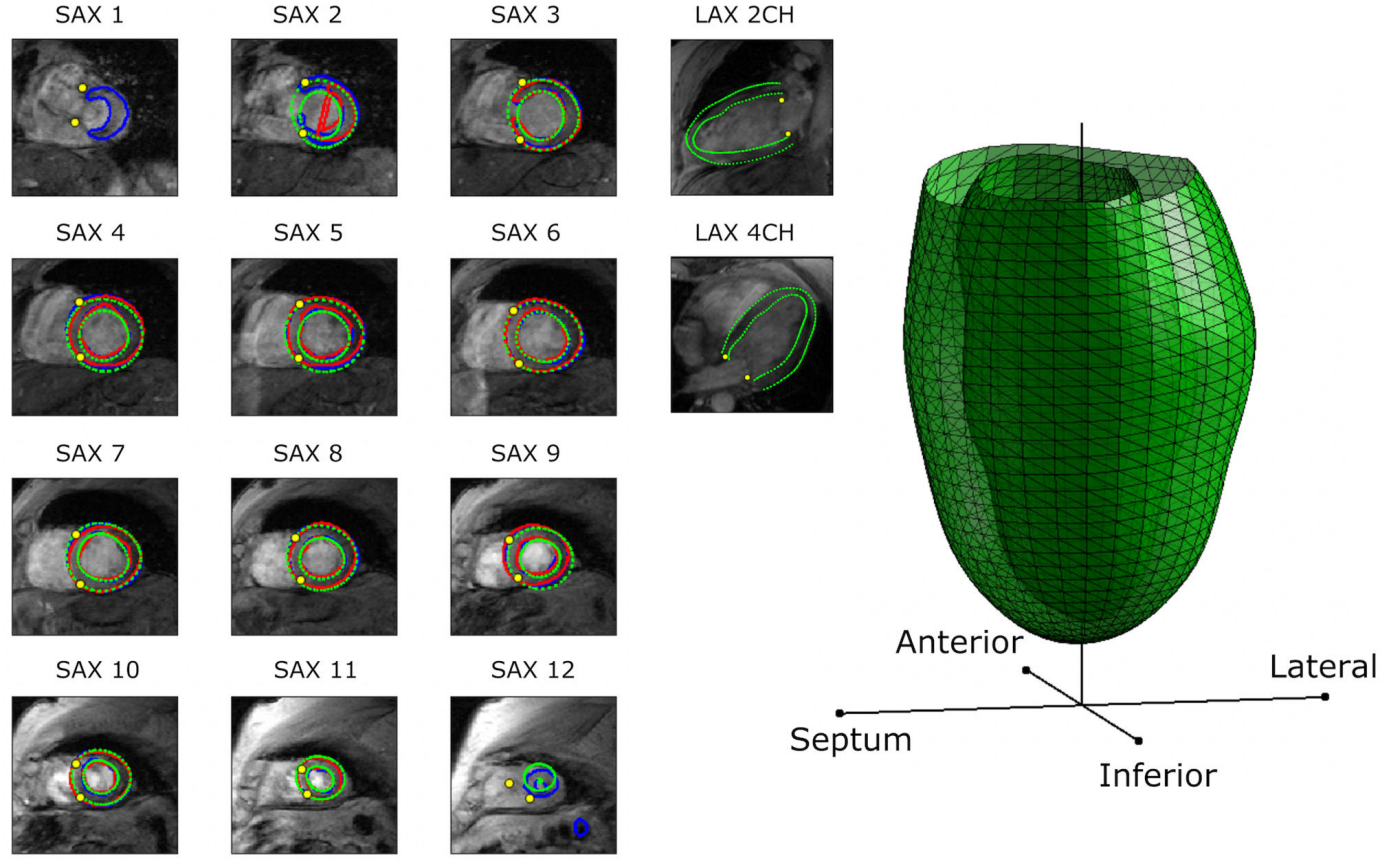
An example of fully automated CMR pipeline result as a patient-specific LV model. Intermediate predictions of the myocardial contours (in blue) and landmark points (yellow circles) are shown in each corresponding DICOM image. Manual contours are shown in red. The intersection contours between the 3D LV model with the images are shown in green. This particular example demonstrates how failed segmentation contours (in apex and base slices) do not affect the final LV model, which are clearly shown in the LAX intersection contours.

For the segmentation task, we demonstrated that the popular U-Net architecture (22) without any major modifications is capable of providing acceptable segmentation of the myocardium in gradient echo cine images. The segmentation network, which was trained based only on individual SAX images (without temporal information), has already achieved excellent performance. The first quartiles of the Dice score were all above 0.85 (Table 4), and 92% of the Dice scores were above 0.80. From the test dataset, the network only failed to segment one slice and only 8 images with Dice scores <0.5. All of these slices were the apical slices, where blood cavity is hardly recognizable even by visual inspection. Other problematic slices were at the base around the outflow tract, where there are more variability of the contours at the aortic root. Figure 5 shows some examples of the segmentation results at different levels of the LV (base, middle and apex) with variations of the Dice scores. Although apical and basal slices were more difficult for the network, the LV shape customization method was relatively robust to segmentation mask outliers, as evidenced by the agreement in statistical relationships with common risk factors, since the model customization process used data from all slices. Figure 7 demonstrates the benefit of the LV model customization over large errors predicted in some problematic slices. This is shown by the intersection contours of the LV model with the SAX images that are well aligned with the manual contours. Figure 7 also shows the intersection contours on LAX images where the alignment of the contours at the myocardium can be visually assessed.

It is known that different groups annotate cardiac MRI data differently (28). For this study, the manual contours were performed by a single core lab, whereas the landmarks were performed in another core lab, so both the landmark detection and segmentation networks will reflect the core lab standard operating procedures on the gradient echo images. Differences in local shape are expected when comparing the shape models generated with gradient echo imaging with those generated from other protocols, and these can be corrected using atlas-based methods (29). Alternatively, the training data distribution can be made richer to include more pathologies, images from different centers and multiple observers, as has been demonstrated by Tao et al. (30) and Bhuva et al. (31).

A common approach to train a complex deep learning network is by end-to-end training (32, 33), where a combined loss function is defined for multiple tasks as the global cost function to optimize. In this work, landmarks and contours were only available on separated image views, so we decided to train the landmark detection network separately to the segmentation network to make each network capable of predicting unseen images independently. The ability to identify mitral valve points therefore does not need to depend on the segmentation masks or vice versa.

The problem of missing information is common to legacy datasets such as MESA. In this study, information linking contours with the corresponding 3D image position was not available. Since most cases were able to be matched with a simple algorithm, leading to sufficient training data, we did not invest more time in developing more sophisticated image-contour matching algorithms. The 3D conversions failed mainly due to missing 3D position information in the DICOM header or missing trigger time information needed to sort the images temporally. To investigate whether there was any bias due to poor image quality, we examined the image quality score given by the original Core Lab readers. This was a three-level subjective rating: 1 for good, 2 for moderate and 3 for poor. There were no significant differences between included and excluded cases (*p* = 0.4, Fisher's Exact test), with 85.4% vs. 86.3% having score 1, 14.6% vs. 13.5% for score 2 and 0% vs. 0.2% for score 3, for included vs. excluded cases, respectively. LV wall motion was also scored on a three point scale and there were no differences between included and excluded cases.

Although this study specifically trained deep learning networks for old legacy gradient echo (GRE) cine images, there are some clinical applications employing GRE imaging. In a recent guideline (34), the image quality of GRE images is better than that of the current steady-state free precession (SSFP) cine images for patients with cardiac implantable electronic devices (35). GRE images are also preferred for T1 and T2-weighted images particularly for patients with suspected iron overload (36). Hence the proposed CMR analysis pipeline has a wider application in other cardiac imaging studies as well, albeit transfer learning is needed to adapt the learned weight parameters to specific pathology. Note that the pipeline does not depend only on GRE; it can be applied directly to other types of CMR images, particularly where legacy datasets can provide valuable additional data.

## Conclusions and Future Work

We have shown that deep learning networks can be used for automatically finding LV landmarks and segmentations on legacy MESA CMR images, in order to automate the construction of LV models, which can be used to build an atlas and evaluate associations between LV shape and risk factors. The final prediction of the LV model based on deep learning networks had similar power to evaluate associations with cardiovascular risk factors compared to manual analysis. This has greatly reduced the amount of time to analyze large-scale collections of cardiac MRI study. In future work, the automated atlas will be used to derive associations between LV shape and outcomes. In addition, analysis of all frames in the cine will allow the calculation of ejection and filling rates and other dynamic information.

## Data Availability Statement

The datasets analyzed for this study are available on request from the Cardiac Atlas Project (www.cardiacatlas.org). Codes will be available from https://github.com/orgs/CardiacAtlasProject/repositories.

## Ethics Statement

The studies involving human participants were reviewed and approved by Johns Hopkins University School of Medicine (NA 00031350) and New Zealand Multiregion Ethics Committee (MEC/08/04/052). The patients/participants provided their written informed consent to participate in this study.

## Author Contributions

AS, CM, BA-V, KG, and AY designed the overall study and performed the final analysis. MJ developed, trained, and validated the landmark detection network. LSH, JDG, and CWe developed, trained, and validated the segmentation network. KG and CM developed and validated the left ventricular fitting method. AS, KG, CM, and AY processed the pipeline, applied the trained networks into the remaining MESA cohort, and performed the independent atlas validation. BA-V, JL, CWu, and DB assessed the final validation results. All authors participated in the analysis, interpretation of data, drafting of the manuscript, revising it critically, and final approval of the submitted manuscript.

## Funding

This research was funded by the Health Research Council of New Zealand (17/608 and 17/234). MESA and the MESA SHARe project are conducted and supported by the National Heart, Lung, and Blood Institute (NHLBI) in collaboration with MESA investigators. Support for MESA was provided by contracts N01-HC-95159, N01-HC-95160, N01-HC-95161, N01-HC-95162, N01-HC-95163, N01-HC-95164, N01-HC-95165, N01-HC-95166, N01-HC-95167, N01-HC-95168, N01-HC-95169, and CTSA UL1-RR-024156.

## Author Disclaimer

The views expressed in this manuscript are those of the authors and do not necessarily represent the views of the National Heart, Lung, and Blood Institute; the National Institutes of Health; or the U.S. Department of Health and Human Services.

## Conflict of Interest

The authors declare that the research was conducted in the absence of any commercial or financial relationships that could be construed as a potential conflict of interest.

## Publisher's Note

All claims expressed in this article are solely those of the authors and do not necessarily represent those of their affiliated organizations, or those of the publisher, the editors and the reviewers. Any product that may be evaluated in this article, or claim that may be made by its manufacturer, is not guaranteed or endorsed by the publisher.
