# Supplementary material for: Deep Learning Analysis of Cardiac MRI in Legacy Datasets: Multi-Ethnic Study of Atherosclerosis
Source: Front Cardiovasc Med. 2022 Jan 21;8:807728. doi: 10.3389/fcvm.2021.807728 (PMC8813768; doi:10.3389/fcvm.2021.807728)
Supplement: Supplementary file 1 [file Presentation_1.pdf]

Appendixes

A. Landmark Detection Network

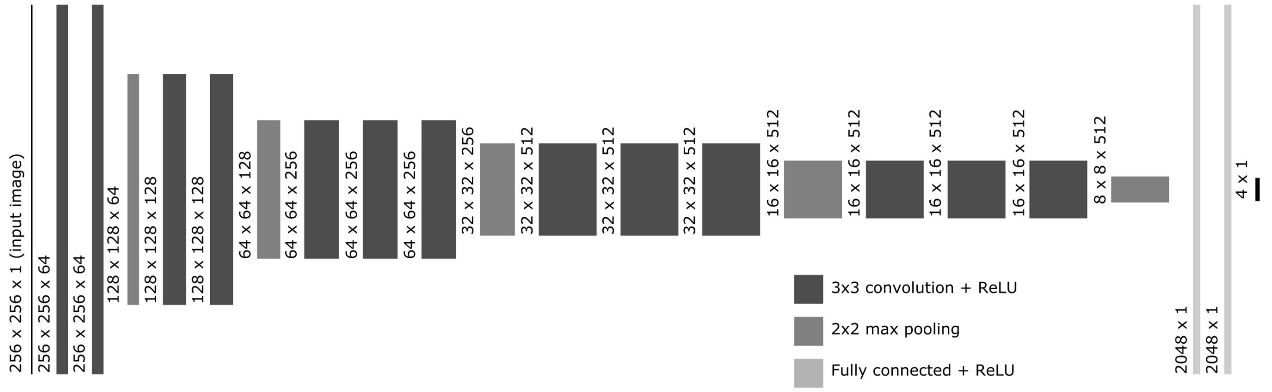

Figure 1. Landmark detection deep learning network architecture based on VGG16.

The landmark detection network was based on the VGGNet architecture (1), which has been successfully used to classify images and to recognize objects. We used the 16-layer VGGNet and trained the network from scratch. We did not use a pre-trained network as a base, because of significant differences between CMR images compared to natural RGB images. We modified the network by reducing the number of neurons from 4096 to 2048 and the size of the final layer was changed to 4 neurons, corresponding to the two cardiac points to be annotated, i.e., pairs of (x, y) image coordinates. The network was trained using a uniform random initialization without temporal or other spatial information. Since two-chamber, four-chamber and short-axis views have distinctive appearances, we trained three separate networks independently, instead of sharing the weights.

The landmark detection network was implemented using Keras with the TensorFlow backend [20], AdaDelta optimizer for backpropagation, initial learning rate of 1.0, $E = 10^{-8}$ and $\rho = 0.95$, and a mini-batch size of 48. On stagnation of the validation loss for three consecutive epochs, the learning rate was reduced by a factor of 5. The network was deemed to be converged when the root mean square error of the normalized coordinates did not decrease by at least 10^{-6} in six consecutive epochs. With the exception of data-augmentation, no additional regularization was performed.

To prevent overfitting and to increase generalization, data augmentation was applied at training time. Images were randomly rotated up to 45 degrees in either direction, shifted by up to 25 pixels in both x and y direction and sheared by a factor between −0.2 and 0.2. The four-chamber and two-chamber were randomly flipped horizontally or vertically respectively, with a probability of 0.3, while the short axis images were randomly scaled with a factor between 0.8 and 1.2.

B. Segmentation Network

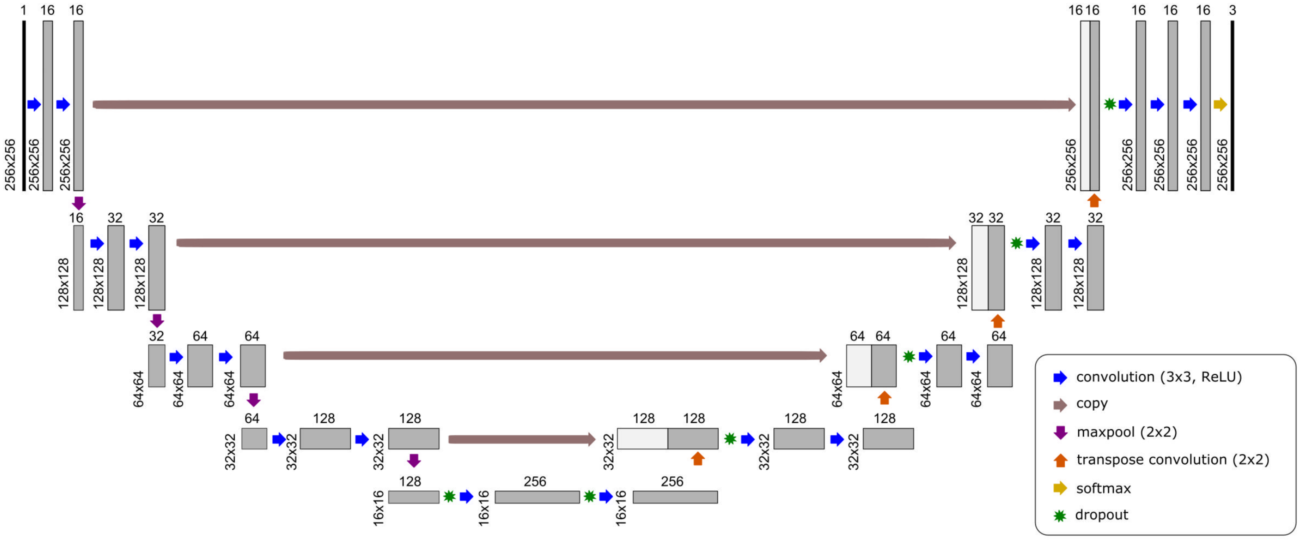

Figure 2. Segmentation deep learning network architecture based on U-Net.

We used a U-Net architecture (2) to segment the myocardium. Briefly, the network consisted of a contracting path and an expansive path. The contracting path consisted of a repeated application of two 3×3 convolutions with batch normalization, each followed by zero-padding, a ReLU and a 2×2 max pooling operation with stride 2 for downsampling. Every step in the expansive path consisted of an upsampling of the feature map followed by a 2×2 convolution with batch normalization, followed by zero-padding and a ReLU. This was followed by a concatenation with the corresponding feature map from the contracting path, and two 3×3 convolutions with batch normalization, each followed by zero-padding and a ReLU. At the second last layer, a 3×3 convolution was performed followed by zero-padding and a ReLU, and at the final layer a 1×1 convolution was used to map each 16-component feature vector to the desired number of three classes. In total the network had 24 convolutional layers. The loss function was computed by a pixelwise softmax cost function over the final feature map combined with the dice loss function.

Images were normalized to have a zero mean and a unit standard deviation. Data augmentations were performed by rotation, random horizontal / vertical image flip, zoom, brightness, and contrast. For rotation augmentation, images were randomly rotated with an angle between 0 and 360 degrees. Bilinear interpolation was applied for the original MR images, while nearest neighbor interpolation was used for the mask images. For the zoom augmentation, the MR images were resized by a scalar of 3 to 8 using bicubic interpolation, and the same operation was applied for the image mask with nearest neighbor interpolation. The brightness/contrast augmentation randomly adjusted the brightness/contrast of the MR images by up to ±50%.

The initial weights of the layers in the segmentation network were random values, which were then optimized by stochastic gradient descent. The training optimization was dependent on the learning rate and loss function. The initial learning rate was 0.001 and was kept at this value for the first ten epochs, after which it was reduced by the square-root of two after every fifth epoch. The loss was calculated as 1 minus the weighted DSC for the myocardium and cavity, which was averaged for the slice.

References:

1. Simonyan K, Zisserman A. Very Deep Convolutional Networks for Large-Scale Image Recognition. In: Bengio Y, LeCun Y, editors. *3rd International Conference on Learning Representations, ICLR 2015, San Diego, CA, USA, May 7-9, 2015, Conference Track Proceedings*. (2015) http://arxiv.org/abs/1409.1556

2. Ronneberger O, Fischer P, Brox T. "U-Net: Convolutional Networks for Biomedical Image Segmentation.," In: Navab N, Hornegger J, Wells WM, Frangi AF, editors. *Medical Image Computing and Computer-Assisted Intervention – MICCAI 2015*. Cham: Springer International Publishing (2015). p. 234–241 doi: 10.1007/978-3-319-24574-4_28
